# Multifunctional silica nanocomposites prime tumoricidal immunity for efficient cancer immunotherapy

**DOI:** 10.1186/s12951-021-01073-2

**Published:** 2021-10-18

**Authors:** Linnan Yang, Feng Li, Yongsheng Cao, Qiang Liu, Guoxin Jing, Jintong Niu, Feiyue Sun, Yechang Qian, Shilong Wang, Ang Li

**Affiliations:** 1 Research Center for Translational Medicine at East Hospital, School of Life Science and Technology, Tongji University, Shanghai, People’s Republic of China; 2 Department of Respiratory Disease, Baoshan District Hospital of Integrated Traditional Chinese and Western Medicine, Shanghai, People’s Republic of China; 3 The Second Department of Urology, Anhui Provincial Children’s Hospital, Hefei, People’s Republic of China; 4 Central Laboratory, First Affiliated Hospital, Anhui Medical University, Hefei, People’s Republic of China

**Keywords:** miR-125a, Dendritic mesoporous silica nanoparticles, Polyethyleneimine, Tumor immune microenvironment, Immune activation

## Abstract

The tumor immune microenvironment (TIME) has been demonstrated to be the main cause of cancer immunotherapy failure in various malignant tumors, due to poor immunogenicity and existence of immunosuppressive factors. Thus, establishing effective treatments for hostile TIME remodeling has considerable potential to enhance immune response rates for durable tumor growth retardation. This study aims to develop a novel nanocomposite, polyethyleneimine-modified dendritic mesoporous silica nanoparticles loaded with microRNA-125a (DMSN-PEI@125a) to synergistically enhance immune response and immunosuppression reversion, ultimately generating a tumoricidal environment. Our results showed that DMSN-PEI@125a exhibited excellent ability in cellular uptake by murine macrophages and the cervical cancer cell line TC-1, repolarization of tumor associated macrophages (TAMs) to M1 type in a synergistic manner, and promotion of TC-1 immunogenic death. Intratumor injection of DMSN-PEI@125a facilitated the release of more damage-related molecular patterns and enhanced the infiltration of natural killer and CD8^+^ T cells. Meanwhile, repolarized TAMs could function as a helper to promote antitumor immunity, thus inhibiting tumor growth in TC-1 mouse models in a collaborative manner. Collectively, this work highlights the multifunctional roles of DMSN-PEI@125a in generating an inflammatory TIME and provoking antitumor immunity, which may serve as a potential agent for cancer immunotherapy.

**Supplementary Information:**

The online version contains supplementary material available at 10.1186/s12951-021-01073-2.

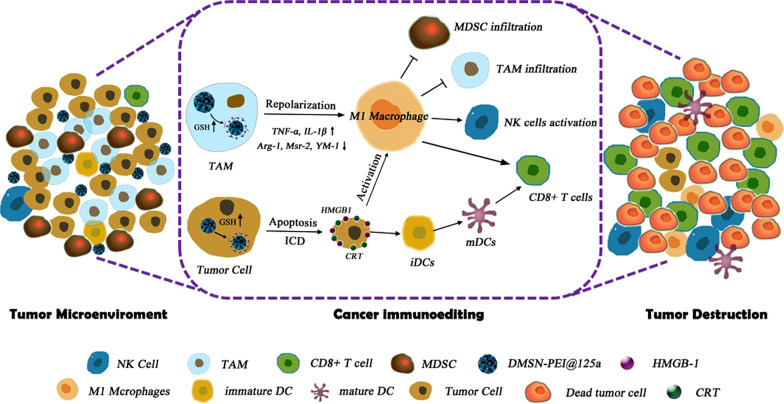

## Background

Malignant tumors remain a primary public health concern, which result in psychological trauma and economic losses to numerous families worldwide [1]. Nowadays, cancer immunotherapy has emerged as a promising strategy for various tumors [2, 3]. However, limited efficiency in solid tumors remains a major issue due to immune dysfunction in the tumor microenvironment, which has evolved gradually with the support of tumor cells [4]. Ideal antitumor immunity needs to satisfy two conditions simultaneously: (1) immunogenic attack of tumor cells and recruitment of immune cells to the tumor site, and (2) remolding of the immunosuppressive tumor microenvironment (ITM) to enhance the immune effector cell infiltration. However, effectively meeting these requirements remains a challenge [5].

It has been established that under continual re-education by tumor cells, immune effector cells lose normal function and become incapable in the tumor immune microenvironment (TIME), by way of: (1) T cell dysfunction [6, 7], (2) impediment of natural killer (NK) cell infiltration and proliferation [8, 9], and (3) macrophages, which promote tumor growth after repolarization by tumor factors [10, 11]. Numerous studies show that macrophages are the most abundant immune cells in TIME, which regulates tumor progression at each stage [12]. Tumor associated macrophages (TAMs), mainly M2 type, unlike M1 macrophages which suppress tumor growth by upregulating IL-12, iNOS and TNF-*α*, express higher levels of immunosuppressive cytokines such as arginase-1 (Arg-1), TGF-*β* and IL-10, inhibit antitumor immunity by recruiting myeloid-derived suppressor cells (MDSCs) and repressing CD8^+^ T cells, and promote the growth and metastasis of tumor cells, which have enabled TAMs to be attractive targets in ITM remodeling [13, 14]. Among multiple TAM-targeting approaches, repolarizing TAM from tumorigenic M2 type to antitumor M1 type exhibits massive potential, generating effector cell infiltration in TIME [15].

Furthermore, several anti-cancer drugs can kill tumor cells while activating the immune system, which is termed as immunogenic cell death (ICD) [16, 17]. Furthermore, immune activation will gradually strengthen with remolding of the immunosuppressive microenvironment [18, 19]. Therefore, combination therapy that induces ICD and reverses ITM, thereby recruiting effective immune cells to aggregate at the tumor site, has great significance in reconstructing a tumoricidal immune microenvironment and accelerating tumor growth inhibition [20, 21].

MicroRNA-125a (miR-125a), a type of non-coding RNA that regulates gene expression by binding to the 3′-untranslated region of targeted mRNA, has demonstrated as an effective modulator in several biological processes, such as cell proliferation and immune cell function regulation [22]. According to previous reports, TAM reprogramming with miR-125a transfection could recover tumoricidal function and facilitate tumor regression [23–25]. In addition, miR-125a exhibits promising applications in tumor growth suppression, such as in cervical carcinoma, breast cancer, melanoma, and colon cancer [26–29]. Hence, miR-125a may serve as an effective gene drug to reverse TAM polarity within ITM and elicit tumor cell death, which accelerates adaptive immune system activation, ultimately restoring the antitumor potential of TIME. Nevertheless, naked gene drugs are easily metabolized and enzymatically digested in vivo [30, 31]; therefore, to construct a new delivery system that effectively improves the bioavailability of gene drugs is the need of the hour.

With the development of nanomedicine, numerous nano-delivery systems have been prepared and applied extensively in cancer immunotherapy [32–35]. Dendritic mesoporous silica nanoparticles (DMSNs) with a center-radial mesoporous silica structure demonstrated by large pore size, volume, and high surface areas, have emerged as a promising instrument for use as nano-vectors [36]. Polyethyleneimine (PEI), a cationic polymer, shows remarkable ability as a gene vector. Meanwhile, other studies have indicated that PEI could skew TAM polarity to the M1 type and suppress tumor progression by elevating proportion of Th1 and NK cells in tumor models [37, 38]. Therefore, it is plausible that fabrication of DMSN-based nanoparticles with PEI may bring many advantages, such as: (1) loading additional payloads compared to naked DMSN particles, (2) protecting gene drug stability from enzymatic degradation, and (3) serving as a functional gene vector by reprogramming TAM to the M1 type.

In this study, based on the large pore size of DMSNs, we prepared a smart nano-vector by connecting PEI via a disulfide bond called DMSN-PEI, realizing drug release in a redox-responsive manner. After DMSN-PEI was loaded with miR-125a as a nanocomposite (DMSN-PEI@125a), we sought to explore the function of DMSN-PEI@125a in remolding ITM in a synergetic manner. Therefore, the main objectives of this study were to: (1) certify the RNase A protection ability and redox-responsive miR-125a release of DMSN-PEI@125a, (2) evaluate the synergistic effect of DMSN-PEI and miR-125a in reversing protumoral M2-like TAMs to antitumor M1 macrophages, (3) confirm the ability of DMSN-PEI@125a to induce apoptosis and immunogenic death of TC-1 cells, and (4) explore the therapeutic effect and the ability to restore tumoricidal immune microenvironment with the joint efforts of TAM repolarization and TC-1 apoptosis and immunogenic death. Finally, we demonstrated that DMSN-PEI@125a could be utilized as a functional agent for cancer immunotherapy.

### Methods

#### Materials

Tetraethyl orthosilicate (TEOS, 98%), cetyltrimethylammonium bromide (CTAB), polyethyleneimine (MW=1800), triethanolamine (TEA), 1-ethyl-3-(3-dimethly aminopropyl) carbodiimide hydrochloride (EDCI), 1-hydroxybenzotriazole monohydrate (HOBT), *N***-**hydroxysuccinimide (NHS), 3-aminopropyltriethoxysilane (APTES), anhydrous *N*,*N*-dimethylformamide (DMF) and 3,3′-dithiodipropionic acid (DTDPA) were obtained from Aladdin Chemistry Co. Ltd. (Shanghai, China). Sodium perfluorooctanoate (PFO) was purchased from Santa Cruz Biotechnology Co. (Dallas, USA). CCK-8 cell counting kit, Annexin V/PI cell apoptosis assay kit, DAPI, and phosphate buffer solution (PBS) were provided by KeyGen Biotech Co. Ltd. (Nanjing, China). Trypsin-EDTA, Dulbecco’s Modified Eagle’s Medium (DMEM; high glucose), RPMI 1640 medium, penicillin-streptomycin solution and fetal bovine serum (FBS) were purchased from HyClone (UT, USA). miR-125a and negative control mimics were synthesized by RiboBio Biotechnology Co. (Guangzhou, China).

### Cells and animals

The murine cervical cancer cell line TC-1 or macrophage cell line RAW264.7, were supplied by the Chinese Academy of Sciences (Shanghai, China). Cells were cultured in DMEM (high glucose) supplemented with 10% FBS and penicillin/streptomycin (50 units mL^−1^) at 37 °C and 5% CO_2_ in a humidified incubator. TAMs were generated using existing laboratory methods. Briefly, cells obtained from the abdomens of C57BL/6J female mice (6–8 weeks) were cultured in RPMI 1640 medium containing 10% FBS, 50 units mL^−1^ penicillin/streptomycin, and 20 ng mL^−1^ recombinant macrophage colony-stimulating factor (M-CSF). Then, the culture supernatant was discarded and fresh medium supplemented with M-CSF (20 ng mL^−1^) and 30% TC-1 culture supernatant was added to maintain for another 3 days once macrophages adhered to the plate. Female C57BL/6J mice (6–8 weeks old) were purchased from the Shanghai Laboratory Animal Co., Ltd. (Shanghai, China) and kept in a pathogen-free animal facility at Tongji University. All animal experimental protocols were authorized by the Institutional Laboratory Animal Resources at the University.

### Synthesis of DMSN

Briefly, a solution containing TEA (68 mg) and ddH_2_O (25 mL) was stirred at room temperature for 30 min, then CTAB (384 mg) and PFO (68.2 mg) were added, and the mixture was stirred at 80 °C for 1 h. Then 4 mL TEOS was rapidly added, and the solution was stirred for another 2 h under the same conditions. The synthesized products were collected and washed with EtOH/ddH_2_O (1:1, v/v) by centrifugation (20,000 rpm, 15 min), then dried thoroughly before calcination at 550 °C for 5 h in air.

### Synthesis of DMSN-PEI

First, the surface was aminated by dispersing DMSNs (100 mg) in isopropanol (120 mL), adding APTES (160 µL), and refluxing for 12 h at 70 °C. The resultant products were collected and washed with anhydrous ethanol by centrifugation (20,000 rpm, 15 min), and then DMSN-NH_2_ were uniformly dispersed in anhydrous DMF. Next, HOBT (0.84 g), EDCI (1.18 g), and DTDPA (1.0 g) were dissolved in 5 mL anhydrous DMF, followed by the dropwise addition of the above DMSN-NH_2_ solution. The mixture was stirred gently at room temperature for 24 h under N_2_ atmosphere. DMSN-s-s-COOH were obtained and washed with anhydrous ethanol by centrifugation (20,000 rpm, 15 min) three times. Lastly, DMSN-s-s-COOH (50 mg), EDCI (0.1 g), and NHS (0.08 g) were dissolved in MES buffer (5 mL, pH 6.5) and stirred for 2 h in dark. Subsequently, PEI (MW=1800, 20 mg mL^−1^, 5 mL) was added to the above solution and stirred for another 24 h at room temperature. DMSN-PEI were collected and washed twice with ddH_2_O by centrifugation (20,000 rpm, 15 min).

### Synthesis of DMSN-PEI@125a

Briefly, DMSN-PEI nanoparticles were formulated as a uniform liquid phase via ultrasound for 30–60 min, then the miR-125a solution was added to obtain DMSN-PEI@125a by shaking at 500 rpm for 30 min at 4 °C. DMSN-PEI@ negative control (DMSN-PEI@NC) and DMSN-PEI@125a-Cy5 were prepared using the same method.

### Characterization

Transmission electron microscopy (TEM) images were obtained using a transmission electron microscope (JEOL, Tokyo, Japan) at 100 kV. The samples were dropped on a copper grid at a concentration of 0.5 mg mL^−1^. Dynamic light scattering (DLS) and zeta potential were measured using a Zetasizer NanoZS Instrument. The samples were dispersed in water at 1.0 mg mL^−1^ concentration and measured at room temperature. N_2_ adsorption-desorption and pore size distributions were measured using a Micromeritics Tristar II system at 77 kV.

### Gel retardation assay

DMSN-PEI@125a composites with different DMSN-PEI to miR-125a mass ratios were prepared. The mass of miR-125a remained constant at 0.4 µg. The naked miR-125a was used as a control. For the loading ratio assay, different samples were loaded on 1% agarose gel in TAE buffer containing GelRed (Beyotime, Jiangsu, China) and subjected to electrophoresis at 100 V for 20 min. The results were visualized using a molecular imaging system (Tanon, Shanghai, China).

### RNase A protection assay

DMSN-PEI@125a containing 0.4 µg miR-125a were incubated with RNase A (4 µg mL^−1^) at 37 °C for 1 h. RNase A was then inhibited, and heparin sodium (50 mg mL^−1^) was added. The solution was incubated for 2 h at 50 °C, and the results were examined by 1% agarose gel electrophoresis.

### Redox-responsive miRNA release

To evaluate the in vitro miR-125a release, DMSN-PEI@125a nanoparticles were suspended in GSH solution (2 × 10^−3^ M or 10^−2^ M) with gentle shaking. At desired time intervals, after centrifugation at 20,000 rpm, the released medium (200 µL) was collected and replenished with an equal volume of fresh medium. The released ratio of miR-125a was measured using a microplate reader.

### Cell viability assay

RAW264.7 and TC-1 cells were seeded in 96-well plates (8 × 10^3^ cells per well) and cultured overnight. Then, different concentrations of DMSN, DMSN-PEI, miR-125a and DMSN-PEI@125a were added and incubated for 24 or 48 h. Cell viability was determined using the CCK-8 assay as previously reported [39].

### Hemolysis assay

The hemolytic activities of DMSN and DMSN-PEI were evaluated as previously described [40]. Briefly, fresh blood collected from C57BL/6J mice was centrifuged and washed several times with PBS solution at 4 °C for 5 min. Red blood cells (RBCs) were obtained and diluted with PBS. Subsequently, DMSN and DMSN-PEI were dispersed in PBS and mixed with the RBC suspension to ensure that the final concentrations of nanoparticles were 20, 40, 80, 160, and 320 µg mL^−1^. RBCs treated with PBS and ddH_2_O were used as negative and positive controls, respectively. Then, the mixtures were vortexed and incubated at room temperature for 2 h. After arriving at the predetermined time, all samples were centrifuged at 12,000×*g* for 5 min. Supernatants were obtained, and OD values were measured at 541 nm using a microplate reader (Molecular Devices, CA, USA).

### Cellular uptake of miRNA assay

TC-1 and RAW264.7 cells were seeded in 35 mm glass bottom dishes (1 × 10^5^ cells per dish) for 24 h, and treated with 50 × 10^−9^ M miR-125a-Cy5 (Ex: 647 nm, Em: 670 nm) or DMSN-PEI@125a-Cy5 (equivalent miR125a-Cy5 concentration: 50 × 10^−9^ M) at different time intervals. At the end of the incubation period, cells were stained with DAPI (Ex: 358 nm, Em: 461 nm). The uptake of miRNAs was evaluated using confocal laser scanning microscopy (TCS SP5II, Leica, Wetzlar, Germany).

### Apoptosis assay

TC-1 cells were seeded in 6-well plates (2 × 10^5^ cells per well) and cultured overnight. DMSN-PEI, DMSN-PEI@NC, DMSN-PEI@125a, and miR-125a were added at the miR-125a/NC concentration of 250 × 10^−9^ M (mass ratio of DMSN-PEI to miR-125a was 25:1) and incubated for 48 h. The apoptosis ratio of each treatment was determined using an Annexin V-FITC/PI apoptosis detection kit according to the manufacturer’s instructions using a BD FACS Verse flow cytometer (NJ, USA).

### Quantitative real time qPCR (RT-qPCR) assay

Total RNA was extracted using TRIzol reagent (Takara, Dalian, China), and reverse transcription was performed using a cDNA transcription kit (Takara). RT-qPCR was performed using SYBR Green mix (Takara) on Light Cycler 96 (Roche, Basel, Switzerland). For mouse *Arg-1*, *TGF-β*, *Msr-2*, *TNF-α*, *IL-1β*,* S100-A8*, *S100-A9* mRNA analysis, *GAPDH* was used as an internal control to normalize the expression level. The primer sequences are shown in Additional file 1: Table S1.

### Western blot assay

Full-length caspase-3, caspase-7, caspase-9 and STAT-3 were purchased from Cell Signaling Technology (MA, USA). TC-1 cells were treated with DMSN-PEI, DMSN-PEI@NC, DMSN-PEI@125a and miR-125a in complete medium for 48 h. Subsequently, cells were collected, and cellular protein lysates were extracted using a total protein extraction kit (KeyGen Biotech). The protein content was determined using the BCA protein assay kit (KeyGen Biotech). Extract (30 µg) was fractionated using SDS-PAGE (12% gel). After that, proteins were transferred to polyvinylidene fluoride membranes (EMD Millipore, MA, USA) at 300 mA for 100 min in an ice bath. After being blocked with tris buffered saline tween (TBST) containing 5% bovine serum albumin for 30–60 min at room temperature, the membranes were incubated with primary antibodies against STAT3, caspase-3, caspase-7, caspae-9, and β-actin antibodies at 4 °C overnight. The bands were visualized using Image Quant LAS4000 mini (GE Healthcare Life Science, NJ, USA) after incubation with HRP-conjugated secondary antibodies.

### Cell types of DMSN-PEI@125a distribution in TC-1 microenvironment

TC-1 tumor models were constructed by injecting TC-1 cells (5 × 10^5^ cells in 100 µL PBS) subcutaneously into the right flank of female C57BL/6J mice (6–8 weeks). Tumor size was calculated according to the following formula: tumor volume = (length × width^2^)/2. When tumors grew to 300–400 mm^3^, mice were i.t. injected with DMSN-PEI@125a-Cy5 at 500 pmol per mouse equivalent  miR-125a-Cy5. After 48 h, tumor tissues were harvested and digested with a collagenase type I (0.05 mg mL^−1^, Sigma-Aldrich, MO, USA) and collagenase type IV (0.05 mg mL^−1^, Sigma-Aldrich) mixture as previously described [39]. The cell suspension was filtered and labeled with mouse anti-CD11b antibody, then cellular uptake of miR-125a-Cy5 was measured via BD FACS Verse flow cytometry.

### Evaluation of tumor volume and biosafety

TC-1 tumors were allowed to grow to a size of 50 mm^3^, and different treatments (ddH_2_O, miR125a, DMSN-PEI@NC, DMSN-PEI@125a) were given via i.t. injection. miR-125a and DMSN-PEI@NC, DMSN-PEI@125a at an equivalent miRNA (miR-125a and negative control) 500 pmol/mouse/time were used to treat every 2 days for six times. Tumor volumes were measured every 3 days using a digital caliper. At the end of the experiment, the serum of each group was extracted for further analysis using a HITACHI 7080 Automatic Biochemical Analyzer (Tokyo, Japan). Major organs (heart, liver, spleen, lung, and kidney) were obtained and sectioned for hematoxylin and eosin (HE) staining. Then the histology of the organs was observed using a light microscope (Olympus, Tokyo, Japan).

### Reversing of TC-1 microenvironment

Mice were sacrificed in 48 h after the final injection, and the tumors were collected and digested as described above. Cell suspensions were labeled with mouse antibodies against CD8-PE, CD45-PE-Cy7, CD11b-FITC, F4/80-APC, Gr-1-PE, NK1.1-FITC, CD3-PE (BioLegend, CA, USA) to identify CD8^+^ T cells (CD8^+^CD45^+^), TAMs (F4/80^+^CD11b^+^), NK cells (NK1.1^+^CD3^−^), and MDSCs (CD11b^+^Gr-1^+^) using flow cytometry. TAMs and MDSCs were sorted using a BD FACS AriaII flow cytometer. The expression levels of M1/M2 markers (iNOS, Arg-1) of TAMs, and S100-A8, S100-A9 of MDSCs were measured by qPCR.

### Data analysis

Data were analyzed by GraphPad software for windows and are shown as mean ± s.d. Significant differences among the groups were determined using one-way ANOVA. *p < 0.05; **p < 0.01; and ***p < 0.001 were considered statistically significant.

## Results and discussion

### Synthesis and characterization of DMSN-PEI

**Fig. 1:**
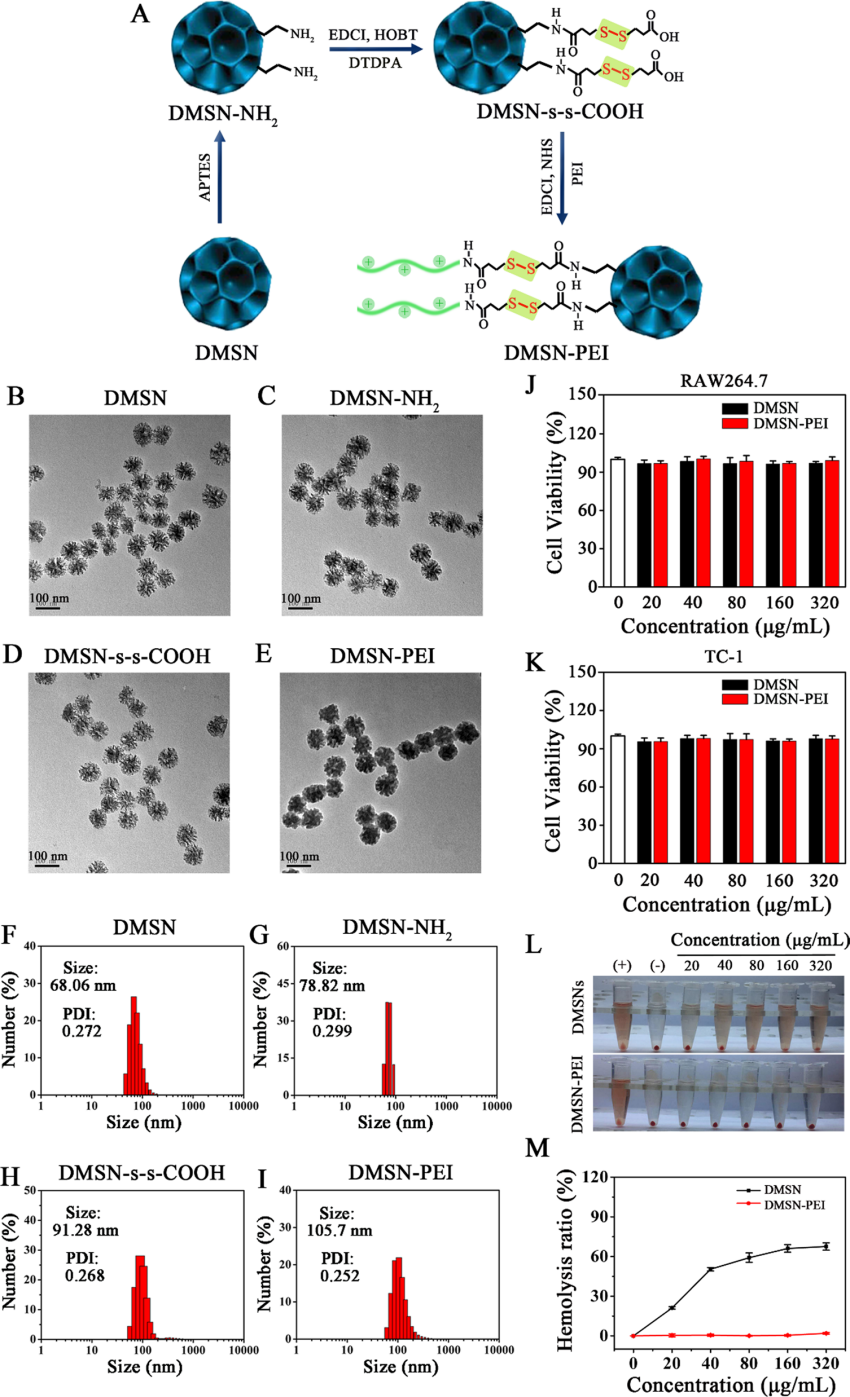
Preparation and characterization of DMSN-PEI. **A** Schematic illustration of the fabrication of DMSN-PEI. **B**–**E** TEM images of **B** DMSN, **C** DMSN-NH_2_, **D** DMSN-s-s-COOH, and **E** DMSN-PEI. Bar = 100 nm. **F**–**I** Particle size distribution of **F** DMSN, **G** DMSN-NH_2_, **H** DMSN-s-s-COOH, and **I** DMSN-PEI determined by DLS. Cytotoxic effect of DMSN and DMSN-PEI against **J** RAW264.7 cells and **K** TC-1 cells for 48 h at different concentrations. Hemolysis assay of DMSNs and DMSN-PEI: **L** digital images and **M** hemolysis percentages. PBS and ddH_2_O treatment were used as negative and positive control, respectively

DMSNs were prepared using the “anionic penetration” method as our previous report [40]. We demonstrated the successful fabrication of DMSN-based multifunctional materials as miRNA carriers. As shown in Fig. 1A, after several steps of the surface modification consisting of amination, carboxylation, and PEI conjugation, we constructed a redox-responsive gene vector via a disulfide linker. TEM images of DMSNs showed spherical morphology and a homogenous 3D dendritic porous shell structure (Fig. 1B). After amino, carboxyl, and PEI decoration, the morphology and size of the particles exhibited no invisible changes compared to naked DMSN (Fig. 1C–E). To ensure redox-responsive fracture of disulfide bonds in DMSN-PEI, the particles were dispersed in GSH solution (10^−2^ M) for 24 h and most pore channels were restored (Additional file 1: Fig. S1), suggesting that adherent PEI could fall off and realize slow release of loaded gene drugs in a GSH-enriched environment. The sizes of various DMSN-based functional materials were measured by DLS. The results indicated that DMSNs had a mean hydrodynamic diameter of approximately 68.06 nm that manifested no clear difference compared with TEM. Moreover, the polydispersity index (PDI) at 0.272 showed high dispersal of the DMSN particles (Fig. 1F). The mean hydrodynamic diameter of DMSN-NH_2_, DMSN-s-s-COOH, and DMSN-PEI were 78.82 nm, 92.28 nm and 105.7 nm, respectively (Fig. 1G–I). All values showed a slight increase after the step-by-step modification.

The results of the zeta potential analysis were presented in Additional file 1: Fig. S2. The zeta potential value for DMSNs was − 14.27 mV. After multistep fabrication, the values of DMSN-NH_2_, DMSN-s-s-COOH, and DMSN-PEI turned to + 2.17, − 23.43, and + 41.6 mV, respectively. These changes indicated the successful decoration at each step during the experiment. The nitrogen sorption isotherm technique was applied to detect the changes in surface areas, pore volumes, and pore sizes before and after DMSNs were decorated by amino, carboxyl, and PEI. DMSNs had a pore volume of 1.4536 cm^3^/g, a BET surface area of 483.2105 m^2^/g, and a broad pore size distribution between 4 and10 nm (Additional file 1: Fig. S3 and Table S2). The surface area and pore volume of DMSNs gradually decreased after each functionalization step. The pore volume of DMSN-PEI reached 0.9888 cm^3^/g, which indicates that DMSN-PEI have a sufficient pore volume to carry gene drugs.

An ideal gene vector should possess good biocompatibility to avoid unnecessary physical side effects. Therefore, the cytotoxicity effect of DMSN and DMSN-PEI were measured in RAW264.7 and TC-1 cells using CCK-8 assay. As depicted in Fig. 1J, K, DMSN and DMSN-PEI exhibited no obvious toxicity to the two cell types after incubation for 48 h, even at treatment concentration up to 320 µg mL^−1^. Moreover, the hemolysis ratio is a key index associated with the safety assessment of particles used for injection [40]. To further investigate the blood compatibility of DMSN and DMSN-PEI, a hemolytic test was performed as previously reported. It is known that the occurrence of hemolysis will cause hemoglobin release from RBCs, and the final obtained solution will turn red. As shown in Fig. 1L, the digital images helped us intuitively understand the hemolysis caused by DMSNs and DMSN-PEI at different concentrations (20, 40, 80, 160, and 320 µg mL^−1^). Compared to the negative control, naked DMSNs resulted in a slight red color of the solution at a concentration of 20 µg mL^−1^, and shades of red deepened with consistent increase. Surprisingly, DMSN-PEI treatment revealed no obvious color changes in the range of 20–320 µg mL^−1^. Subsequently, hemolytic ratios were determined and the result was displayed in Fig. 1M, in terms of DMSNs, after the treatment concentration reached 320 µg mL^−1^, the determined hemolytic ratio was 67.59%. The DMSN-PEI treatment was 0.88% under the same condition, indicating that DMSN-PEI had better hemocompatibility than DMSNs. Collectively, these results indicate that DMSN-PEI have good biocompatibility in vitro and may serve as an ideal gene vector for in vivo application.

### Synthesis, characterization, and cellular uptake of DMSN-PEI@125a in vitro and in vivo

**Fig. 2:**
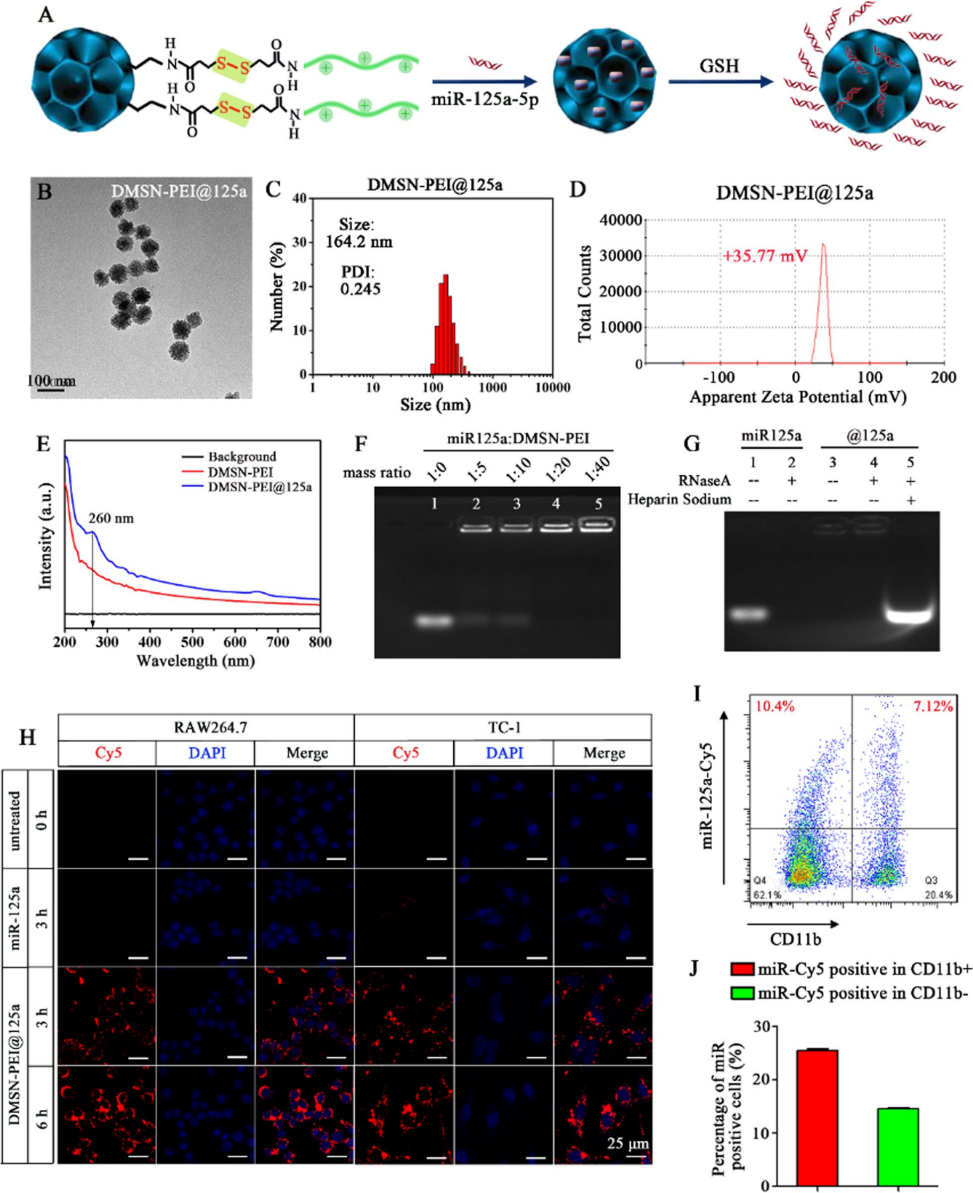
Preparation and characterization of DMSN-PEI@125a nanocomposites. **A** Schematic illustration of DMSN-PEI@125a preparation and redox-responsive release in cells. **B** TEM image of DMSN-PEI@125a. **C** Particle size distribution of DMSN-PEI@125a measured by DLS. **D** Zeta potential of DMSN-PEI@125a (mass ratio of DMSN-PEI to miR-125a is 100:1). **E** UV absorption spectrum of DMSN-PEI and DMSN-PEI@125a. **F** miR-125a loading capacity and **G** RNase A protection assay detected by agarose gel electrophoresis. Cellular uptake of DMSN-PEI@125a and free miR-125a in vitro and in vivo. **H** Fluorescence confocal microscopy images of free miR-125a and DMSN-PEI@125a uptake by RAW264.7 cells and TC-1 cells in at different time points. Bar = 25 μm. **I** Tumor bearing C57BL/6J mice were i.t. injected with DMSN-PEI@125a. The uptake of miR-125a by TAMs (CD11b positive) or tumor cells (CD11b negative) in tumors were detected at 48 h using flow cytometry. **J** Quantitative percentage of miR-125a-Cy5 positive cells in CD11b positive or negative cells

Figure 2A illustrates the DMSN-PEI@125a preparation and redox-responsive release of miR-125a in a GSH-enriched environment. TEM images revealed that DMSN-PEI@125a exhibited a spherical morphology, and the central-radial pore structures became invisible after loading with miR-125a (Fig. 2B). The mean hydrodynamic diameter of DMSN-PEI@125a further increased to 164.2 nm with a low PDI value of 0.245, suggesting that the nanoparticles dispersed well uniformly in the aqueous solution (Fig. 2C). DMSN-PEI@125a (DMSN-PEI to miR-125a mass ratio is 100:1) showed a lower zeta potential at + 35.77 mV compared to naked DMSN-PEI, indicating the successful loading of miR-125a (Fig. 2D). Furthermore, such a positive charge contributed to easy internalization by cell membranes, which presented negative charges, and improved transfection efficiency. The UV spectra of DMSN-PEI and DMSN-PEI@125a were depicted in Fig. 2E, which clearly showed the peak at 260 nm for DMSN-PEI@125a as a result of the existence of miR-125a. The miRNA condensation capability of particle is crucial in nanoparticle-based miRNA delivery. Herein, the saturation of the miR-125a binding to DMSN-PEI was assessed by agarose gel retardation assay. As shown in Fig. 2F, the miRNA condensation ability was measured at different DMSN-PEI to miR-125a mass ratios, and the result manifested that miR-125a was retarded in spotting holes entirely at the mass ratio of 20, suggesting that miR-125a could be carried completely by DMSN-PEI at this mass ratio. To further evaluate whether the central-radial pore structure of DMSN-PEI could protect miR-125a from degradation, an RNase A protection assay was conducted by agarose gel electrophoresis. As shown in Fig. 2G, the results showed that naked miR-125a could be easily degraded upon exposure to RNase A (Lane 2), whereas the bands of DMSN-PEI@125a in spotting holes were stable and bright regardless of the presence or absence of RNase A (Lane 3–4). Meanwhile, miR-125a could be released from DMSN-PEI and appeared at the same band position as the control miR-125a (Lane 1) when heparin was introduced, suggesting that DMSN-PEI possessed the capability to protect miR-125a from RNase A degradation (Lane 5). Moreover, redox-responsive miR-125a release from DMSN-PEI was also confirmed. As shown in Additional file 1: Fig. S4, the release efficiency of miR125a in PBS and PBS with GSH was conducted constantly for 5 h at a 500 rpm stirring speed. The release ratio was approximately 13.9% in PBS solution without GSH. Upon exposure to a GSH-rich environment, the release efficiency of miR-125a was enhanced by up to 36.2%. Moreover, the redox-responsiveness of DMSN-PEI@125a would not only prevent miR-125a release under extracellular conditions, but also accelerate miR-125a release in an intracellular bioreducible environment.

Cellular uptake efficiency of DMSN-PEI@125a and free miR-125a in murine TC-1 and RAW264.7 cells was tested by confocal laser scanning microscope. As depicted in Fig. 2H, untreated cells showed no visible red fluorescence, indicating that the cells themselves had no influence on the red fluorescence intensity. Moreover, DMSN-PEI@125a and miR-125a were incubated with RAW264.7 and TC-1 cells, respectively, for 3 h. As expected, free miR-125a was difficult to enter into cells and showed no detectable fluorescent signal, which was consistent with previous research [30, 31]. It was observed that DMSN-PEI@125a demonstrated clear fluorescent signals in both cell lines, exhibiting superior ability in transfection of cells compared with free miR-125a. Meanwhile, when the incubation time was prolonged to 6 h, the intensity of intracellular red fluorescence was much stronger, suggesting that DMSN-PEI@125a nanocomposites were swallowed by RAW264.7 or TC-1 cells in a time-dependent manner.

Furthermore, we used Cy5-labeled miR-125a to investigate the main cell types participated in internalization of DMSN-PEI@125a in TC-1 microenvironment in vivo. Tumor-bearing mice were i.t. injected with DMSN-PEI@125a for 48 h. As shown in Fig. 2I, J, approximately 25.48% CD11b positive cells (mainly TAMs) while nearly 14.58% CD11b negative cells (mainly tumor cells) were Cy5 positive cells (miR^+^) in tumor suspensions, suggesting that TAMs and tumor cells all served as targets of DMSN-PEI@125a in tumors. Overall, DMSN-PEI can facilitate cellular uptake of miR-125a into macrophages and TC-1 tumor cells either in vitro or in vivo, and enhance cytosolic release of miR-125a in the intracellular bioreducible environment, both of which are essential for gene therapy via miR-125a.

### DMSN-PEI@125a skew TAMs to M1 macrophages and induce TC-1 apoptosis and ICD in vitro

**Fig. 3:**
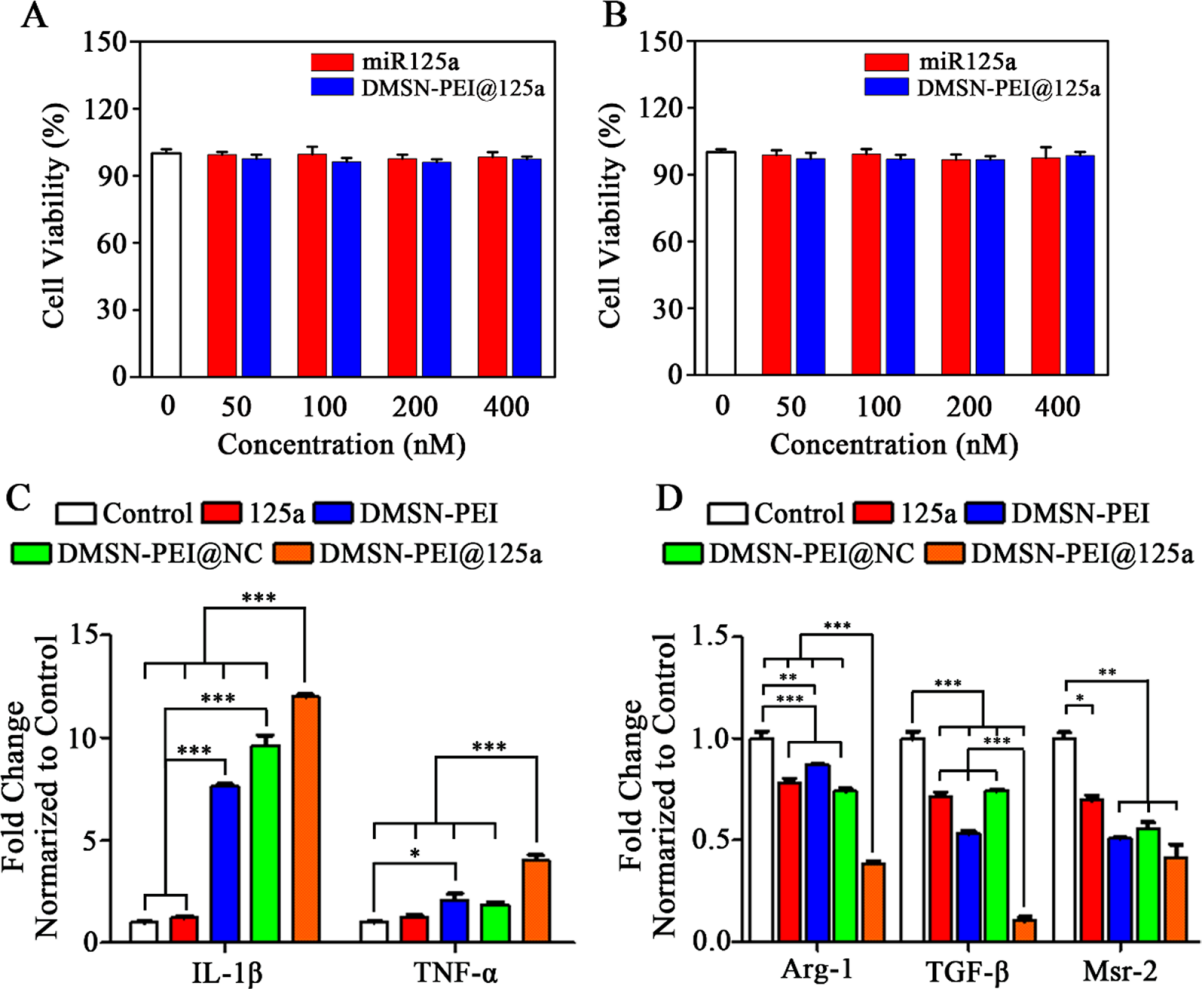
DMSN-PEI@125a induce macrophage phenotype switch in vitro. **A**, **B** Cytotoxic effect of miR-125a and DMSN-PEI@125a against RAW264.7 cells at different concentrations for **A** 24 h and **B** 48 h. **C**, **D** TAMs induced from mouse peritoneal macrophages were treated with DMSN-PEI, DMSN-PEI@NC, DMSN-PEI@125a, and miR-125a for 48 h. The mRNA expression levels of **C** M1 markers (*IL-1β, TNF-α*) and **D** M2 markers (*Arg-1*, *TGF-β*, *Msr-2*) in TAMs, and the fold change are normalized to control group (concentration of miR-125a or NC is 200 × 10^−9^ M, and DMSN-PEI to miR-125a mass ratio is 20:1)

According to a previous report, cationic polymer PEI could reverse TAM repolarization to the M1 type mediated by toll-like receptors [37]. Meanwhile, miR-125a has a similar function as PEI on TAM repolarization [23]. Next, we assessed whether PEI modified on DMSN possessed effective partnerships to create synergies with miR-125a on skewing TAMs to M1 macrophage. First, the cytotoxic effect of DMSN-PEI@125a and free miR-125a on RAW264.7 cells were evaluated using CCK-8 assay. As depicted in Fig. 3A, B, all groups showed no obvious toxicity after 24 or 48 h treatments, even at concentrations up to 400 nM. The effects of DMSN-PEI, DMSN-PEI@NC, DMSN-PEI@125a, and free miR-125a on TAM repolarization were evaluated by detecting the mRNA expression levels of M1 and M2 markers. Mouse peritoneal macrophages were acquired from female C57BL/6J mice and treated with M-SCF cytokines (20 ng mL^−1^) and culture supernatant of TC-1 cells to obtain TAMs. After incubation with the above materials for 48 h, total mRNA of each group was extracted and analyzed using qPCR. As shown in Fig. 3C, D, as we expected, incubation with cargo-free DMSN-PEI increased the expression of M1 markers (*IL-1β*, *TNF-α*) and decreased that of M2 markers (*Msr-2*). Meanwhile, when miR-125a was carried by DMSN-PEI, the M1 marker increase and M2 marker decrease became more prominent, such as elevated *IL-1β* and *TNF-α* levels by 12-fold and 4-fold, respectively, and suppressed *Arg-1*, *TGF-β*, and *Msr-2* expression by 60–90%. However, miR-125a alone did not affect M1 markers expression, but could decrease the expression of M2 markers (*Arg-1*, *TGF-β*, *Msr-2*) slightly due to inability of transfection.

**Fig. 4:**
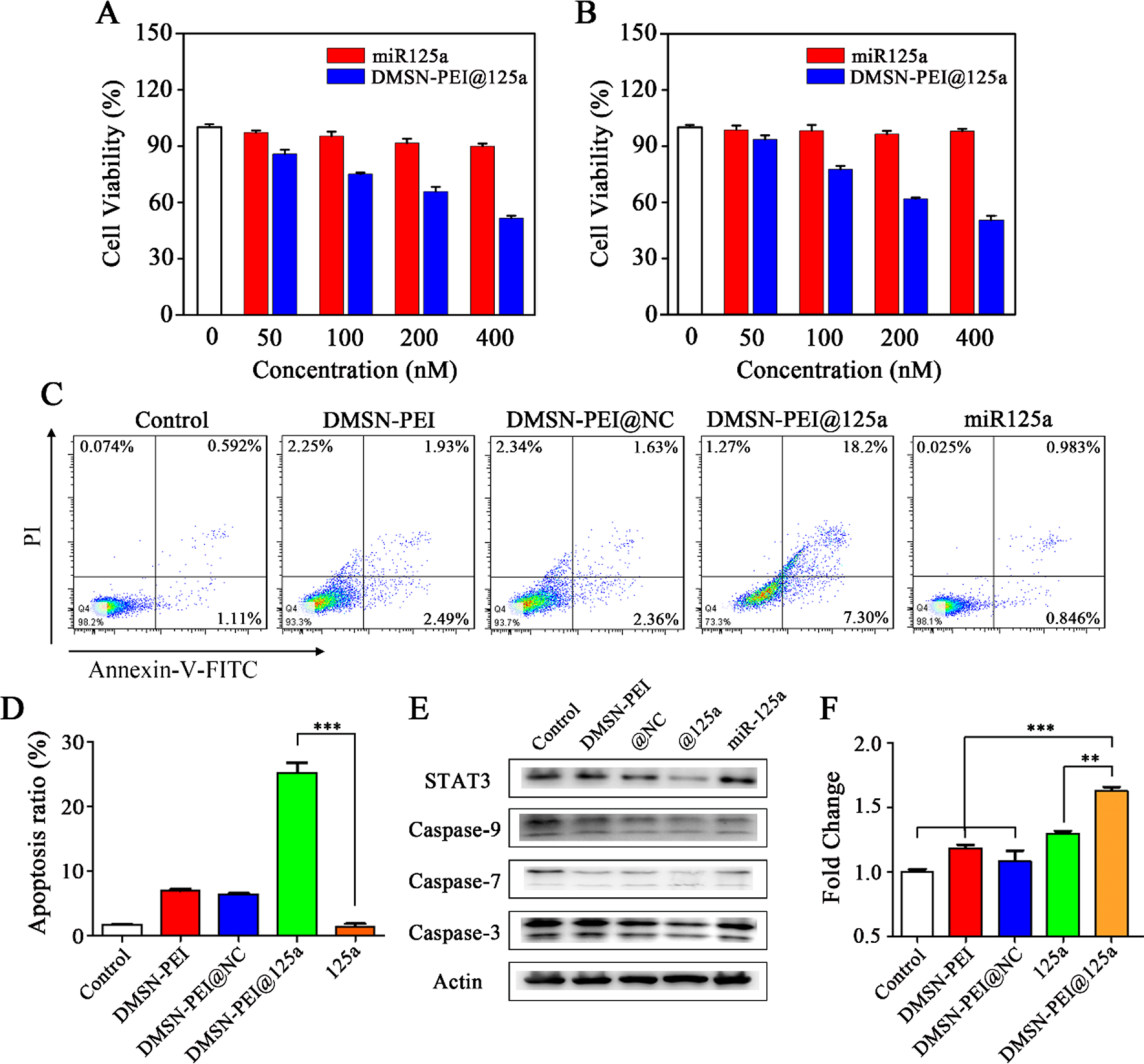
DMSN-PEI@125a induce TC-1 cell apoptosis and immunogenic death in vitro. **A**, **B** Cytotoxic effect of miR-125a and DMSN-PEI@125a on TC-1 cells at different concentrations for **A** 24 h and **B** 48 h. TC-1 cells were treated with DMSN-PEI, DMSN-PEI@NC, DMSN-PEI@125a, and miR-125a for 48 h. (concentration of miR-125a or NC is was 250 × 10^−9^ M, mass ratio of DMSN-PEI to miR-125a is 25:1). **C** Representative flow cytometry images and **D** percentage analysis of TC-1 apoptosis. **E** Expression levels of apoptosis-related proteins (STAT-3, caspase-3, caspase-7, caspase-9) were detected by western blotting. **F** mRNA expression level of calreticulin (CRT) in TC-1 cells was detected using qPCR

miR-125a has the ability to suppress growth in several malignant tumor types, such as cervical, breast, colon, and gastric cancers. We further evaluated whether DMSN-PEI@125a suppressed TC-1 cell proliferation using CCK-8 assay. As shown in Fig. 4A, B, free miR-125a had difficulty in transfecting TC-1 cells, therefore, no obvious influence was found at any of the treatment concentrations. However, DMSN-PEI@125a showed dose-dependent cytotoxicity on TC-1 cells after incubation for 24 or 48 h. In detail, the viability of TC-1 cells treated with DMSN-PEI@125a at equivalent miR-125a concentrations of 50, 100, 200, and 400 × 10^−9^ M were 93.63%, 77.60%, 61.85% and 50.49%, respectively, at 48 h. To further investigate the mechanism by which DMSN-PEI@125a induced toxicity in TC-1 cells, apoptosis experiments were performed using an Annexin V-FITC/PI detection kit by flow cytometry. As depicted in Fig. 4C, D, compared to the control group, TC-1 cells treated by DMSN-PEI or DMSN-PEI@NC showed a negligible apoptosis ratio. miR-125a exhibited no apoptotic effect, which was attributed to the above-mentioned reason. Meanwhile, TC-1 cells treated with DMSN-PEI@125a for 48 h resulted in a total apoptosis ratio of 25.27%, which was far better than other treatments. Furthermore, to further explore the molecular mechanism of TC-1 cell apoptosis induced by DMSN-PEI@125a, apoptosis-related proteins, such as caspase-3, -7, -9 and STAT-3 were determined using western blotting assay. As shown in Fig. 4E, the bands of full-length caspase-3, caspase-7, caspase-9 and STAT-3 were weakened under DMSN-PEI@125a treatment compared with the control, indicating that the caspase and STAT pathways might be involved in DMSN-PEI@125a-induced TC-1 apoptosis. Previous studies corroborated that STAT-3 and caspase-3/9 pathways participated in miR-125a-induced apoptosis of malignant tumor cells. As expected, consistent with the previous findings, our present study revealed that the expression levels of Caspase-3/7/9 and STAT-3 were remarkably suppressed by DMSN-PEI@125a in TC-1 apoptosis.

Recently, drug-induced ICD in malignant tumors has attracted widespread attention. The release of DAMPs induces ICD by recruitment and induction of immune cells, which facilitate immune recognition, phagocytosis of antigen-presenting cells (APCs) and T cell activation to cause tumor regression. DMSN-PEI@125a-induced immunogenic death in TC-1 cells was also verified in our present study. As shown in Fig. 4F, the mRNA expression level of calreticulin (*CRT*), a type of DAMP that recruits and activates APCs by an “eat me” signal in tumor microenvironment, was evaluated by qPCR. We observed that DMSN-PEI@125a treatment contributed to the increase in *CRT* expression, approximately 1.63-fold compared to the control group, indicating that immunogenic death may participate in DMSN-PEI@125a-induced growth inhibition of TC-1 cells.

In conclusion, these results illustrate that DMSN@PEI@125a could reverse TAM polarity to antitumor M1 macrophages in a synergetic manner, facilitate tumor apoptosis and immunogenic death in order to activate effector cells, and potentially repress tumor growth. Therefore, DMSN-PEI@125a may serve as a multifunctional nanocomposite to reshape the TC-1 microenvironment.

### DMSN-PEI@125a can suppress TC-1 growth by remolding the tumor immunosuppressive environment in vivo

**Fig. 5:**
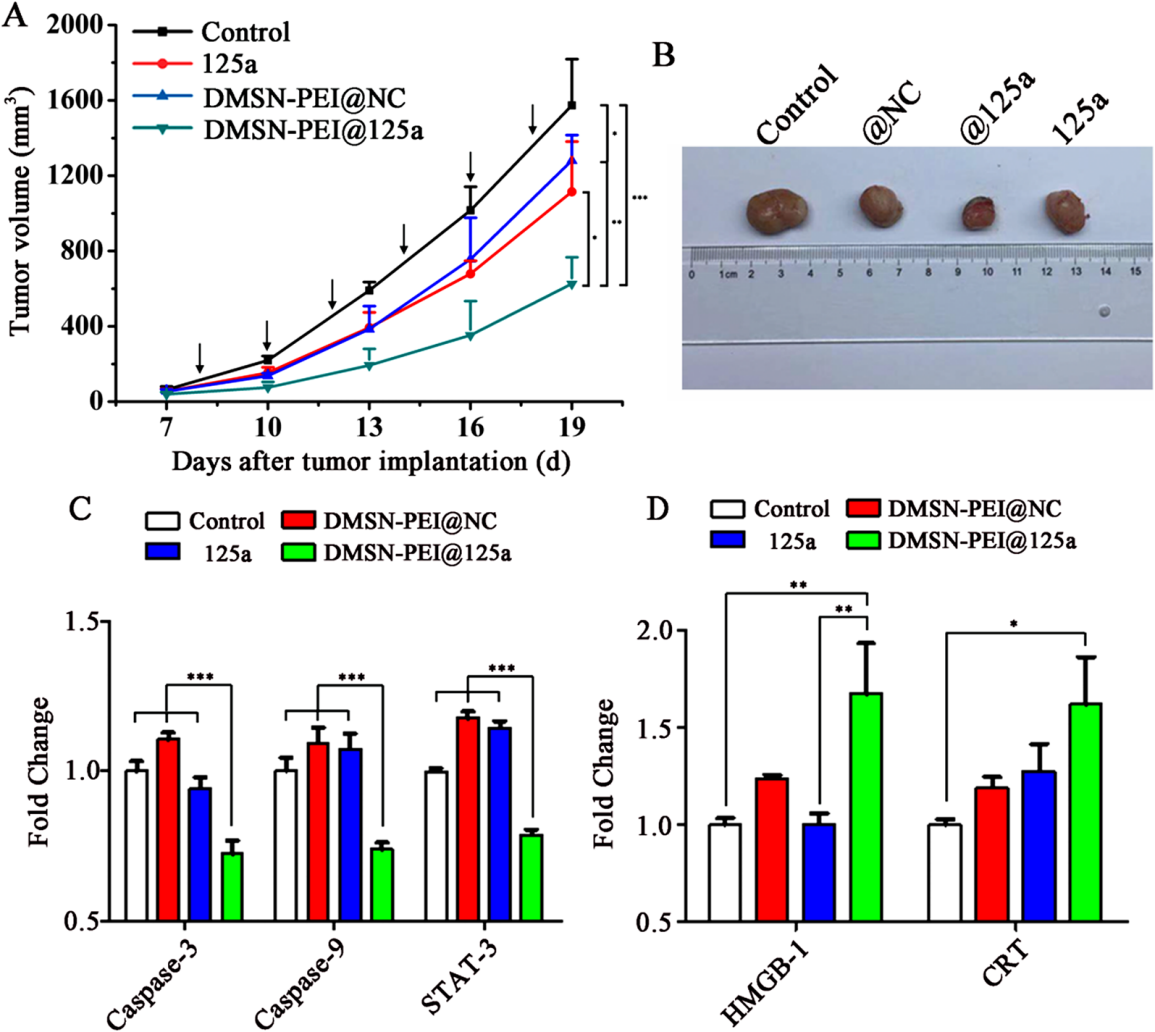
Antitumor effect of DMSN-PEI@125a in vivo. The TC-1 tumor models (n = 5) were i.t. injected with ddH_2_O, DMSN-PEI@NC, DMSN-PEI@125a, and miR-125a every 2 days for six times from day 8 after tumor inoculation. **A** Tumor growth curves of different treatments. Arrows: the day of administration. **B** Photographic images of representative tumors on day 20 after tumor implantation. **C**, **D** mRNA expression levels of **C** apoptosis-related genes (*caspase-3*, *caspase-9*, *STAT-3*) and **D** damage-associated molecular patterns (*HMGB-1*, *CRT*) in tumors

To investigate the therapeutic efficacy of DMSN-PEI@125a in vivo, tumor inhibition experiments were conducted in tumor models of TC-1. Mice were i.t. injected with ddH_2_O, DMSN-PEI@NC, DMSN-PEI@125a, and miR-125a every 2 days for six times from day 8 after tumor inoculation. As can be seen in Fig. 5A, B, DMSN-PEI@NC and miR-125a groups showed a similar tumor-inhibition profile, and the tumor-inhibition rates after six dosages were approximately 18.6% and 29.2%, respectively. Meanwhile, DMSN-PEI@125a inhibited tumor growth more effectively, with a 60.2% reduction in tumor volume compared to that of the control group.

At the end of the experiment, tumors were excised from C57BL/6J mice and mRNA expression levels of apoptosis-related genes (*caspase-3*, *caspase-9*,* STAT-3*) and damage-associated molecular patterns (high mobility group box 1 [*HMGB-1*], *CRT*) in tumors were evaluated using qPCR. As depicted in Fig. 5C, apoptosis-related gene expression in the DMSN-PEI@125a group was suppressed compared to the control, DMSN-PEI@NC, and miR-125a groups, indicating that DMSN-PEI@125a could result in improved TC-1 apoptosis in vivo. Moreover, DMSN-PEI@125a also showed considerable potential in promoting immunogenic death of TC-1 cells. As depicted in Fig. 5D, expression levels of *CRT*, *HMGB-1* improved by 1.67-, and 1.62-fold, respectively, compared to the control group. Exposure of CRT and HMGB-1 in TC-1 cells possibly send “eat me” and “danger” signals to the environment, which attract APCs to swallow and process tumor antigens and form an immunologically activated microenvironment.

**Fig. 6:**
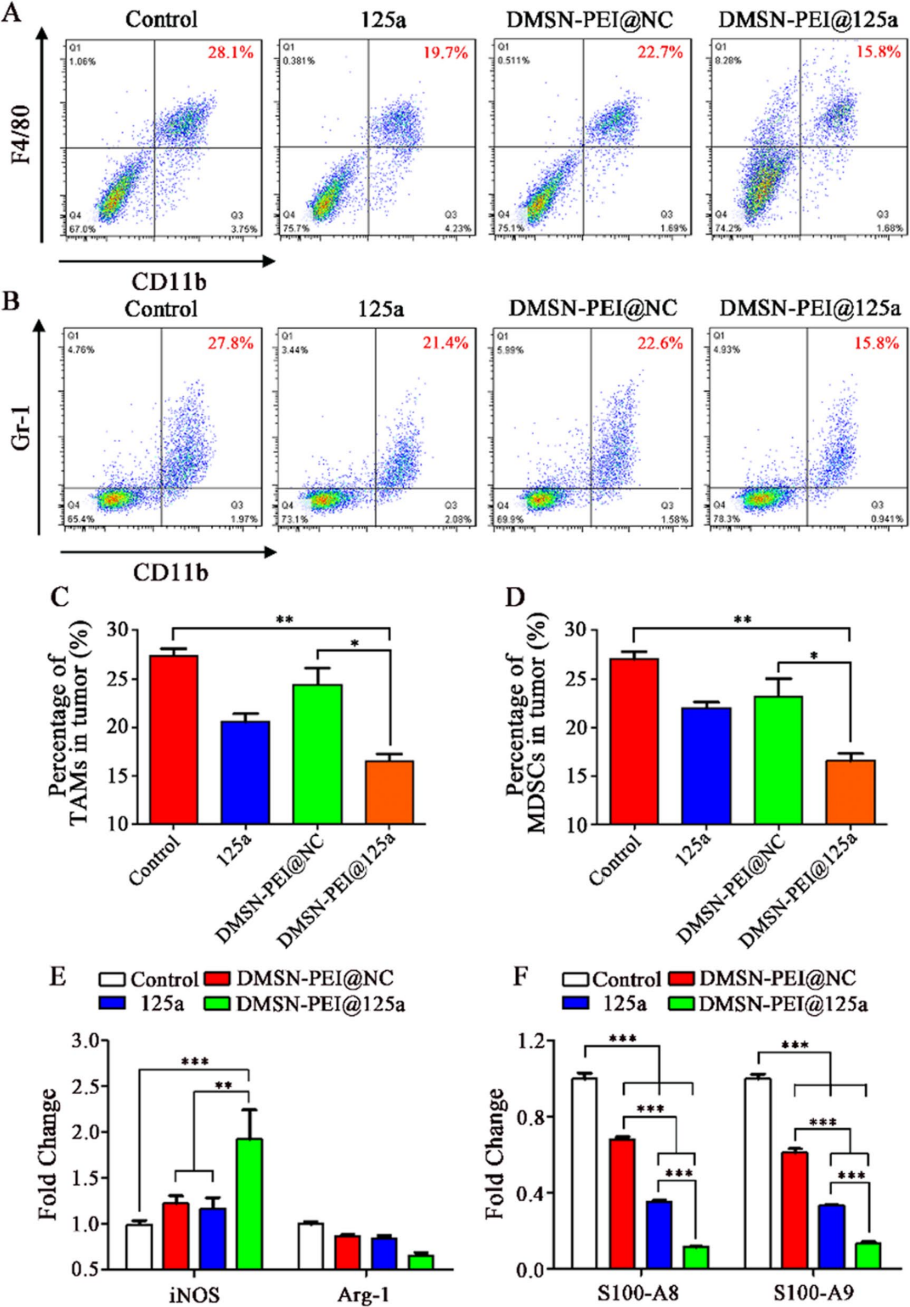
DMSN-PEI@125a suppress TAM (F4/80^+^CD11b^+^) and MDSC (Gr1^+^CD11b^+^) infiltration in TC-1 microenvironment. **A**, **B** Representative FACS images of **A** TAM and **B** MDSC from TC-1 tumors after different treatments. **C**, **D** Infiltration ratio of **C** TAMs and **D** MDSCs in TC-1 tumors after different treatments. **E**, **F** mRNA expression level of **E** M1 marker (*iNOS*), M2 marker (*Arg-1*) in TAMs, and **F** *S100-A8* and *S100-A9* in MDSCs from TC-1 tumors determined using qPCR

**Fig. 7:**
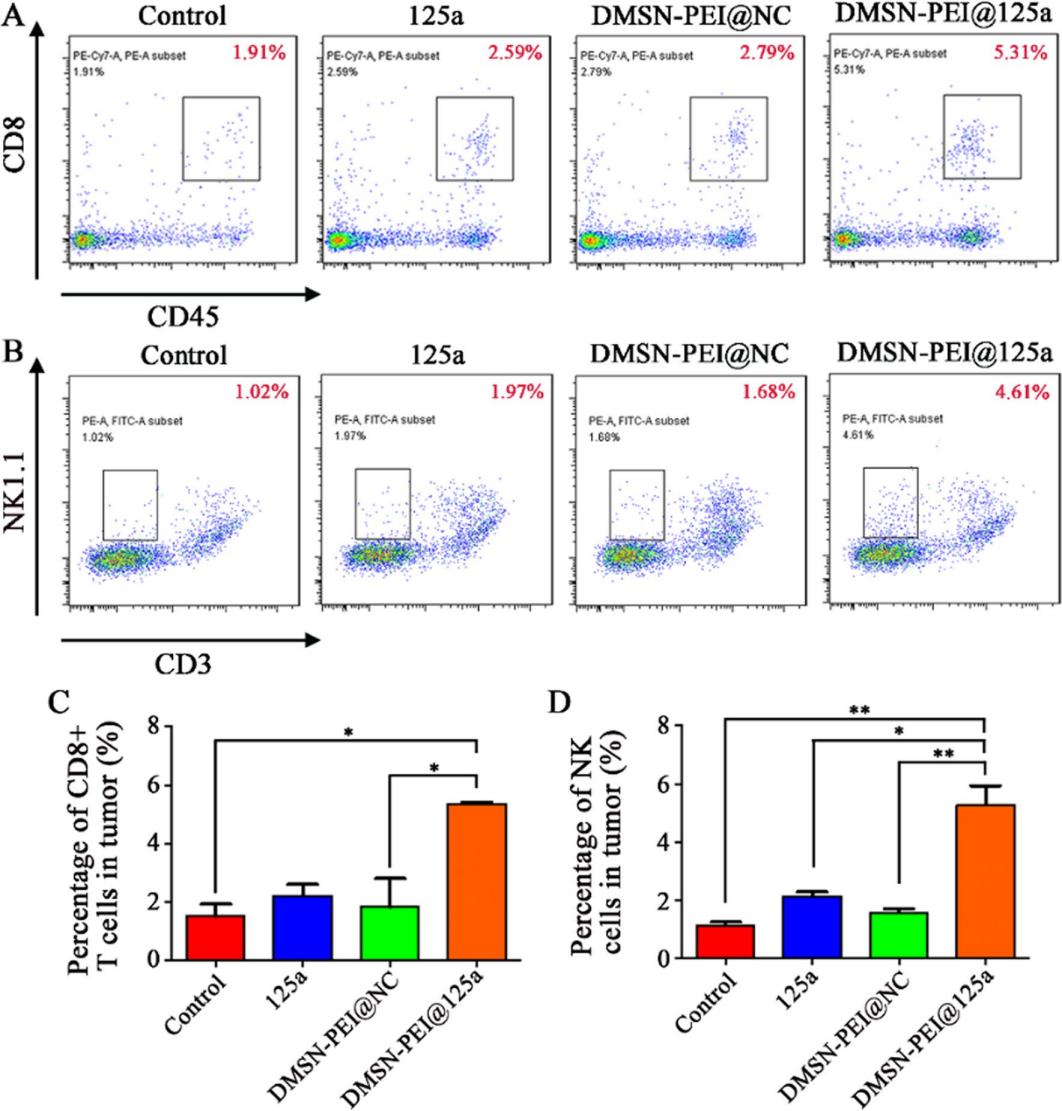
DMSN-PEI@125a facilitate CD8^+^ T cells (CD8^+^CD45^+^) and NK cells (NK1.1^+^CD3^−^) infiltration in TC-1 microenvironment. **A**, **B** Representative FACS images of **A** CD8^+^ T cells and **B** NK cells from TC-1 tumors after different treatments. **C**, **D** Infiltration ratio of **C** CD8^+^ T cells and **D** NK cells in TC-1 tumors after different treatments

The quantity and function of various immune cells in the TIME were evaluated. As shown in Fig. 6, we observed that the infiltration ratio of CD11b^+^F4/80^+^ TAMs (Fig. 6A, C) and CD11b^+^Gr-1^+^ MDSCs (Fig. 6B, D) were remarkably decreased in the DMSN-PEI@125a group, 41% and 38% lower, respectively, than that in the control group. Meanwhile, miR-125a or DMSN-PEI@NC treatment also reduced the number of TAMs and MDSCs to a certain degree; however, their influences were weaker than that of DMSN-PEI@125a. We then sorted TAMs and MDSCs to evaluate the functional changes influenced by DMSN-PEI@125a. As depicted in Fig. 6E, the M1 marker (*iNOS*) expression in the DMSN-PEI@125a group was elevated by 1.93-fold and the M2 marker (*Arg-1*) expression was suppressed by 35% compared with the control, which promoted TAM skew to M1 type. Furthermore, the expression levels of *S100-A8* and *S100-A9* in MDSCs, two types of immunosuppressive genes that recruit MDSCs to TIME were also detected by qPCR. As shown in Fig. 6F, either DMSN-PEI@NC or miR-125a exhibited inhibition abilities against *S100-A8* and *S100-A9* expression in vivo, decreasing *S100-A8* expression by 32% or 65% and *S100-A9* expression by 39% or 66%, respectively. The inhibition effect was further reinforced in the DMSN-PEI@125a group, up to 89% (*S100-A8*) and 87% (*S100-A9*). Repolarized TAMs release a variety of proinflammatory cytokines (e.g., IL-12, iNOS), which could ultimately recruit multiple effector cells (e.g., CD8^+^ T and NK cells) to inhibit tumor progression. Furthermore, upregulated expression of HMGB-1 and CRT could also promote the presentation of tumor antigens to CD8^+^ T cells, resulting in an adaptive immune response. Herein, we measured the infiltration ratio of CD8^+^ T (CD8^+^CD45^+^) and NK cells (NK1.1^+^CD3^−^), respectively. As expected, DMSN-PEI@125a significantly increased the number of CD8^+^ T (Fig. 7A, C) and NK cells (Fig. 7B, D) in TIME compared with the DMSN-PEI@NC and miR-125a groups that exhibited a slight promoting effect.

**Fig. 8:**
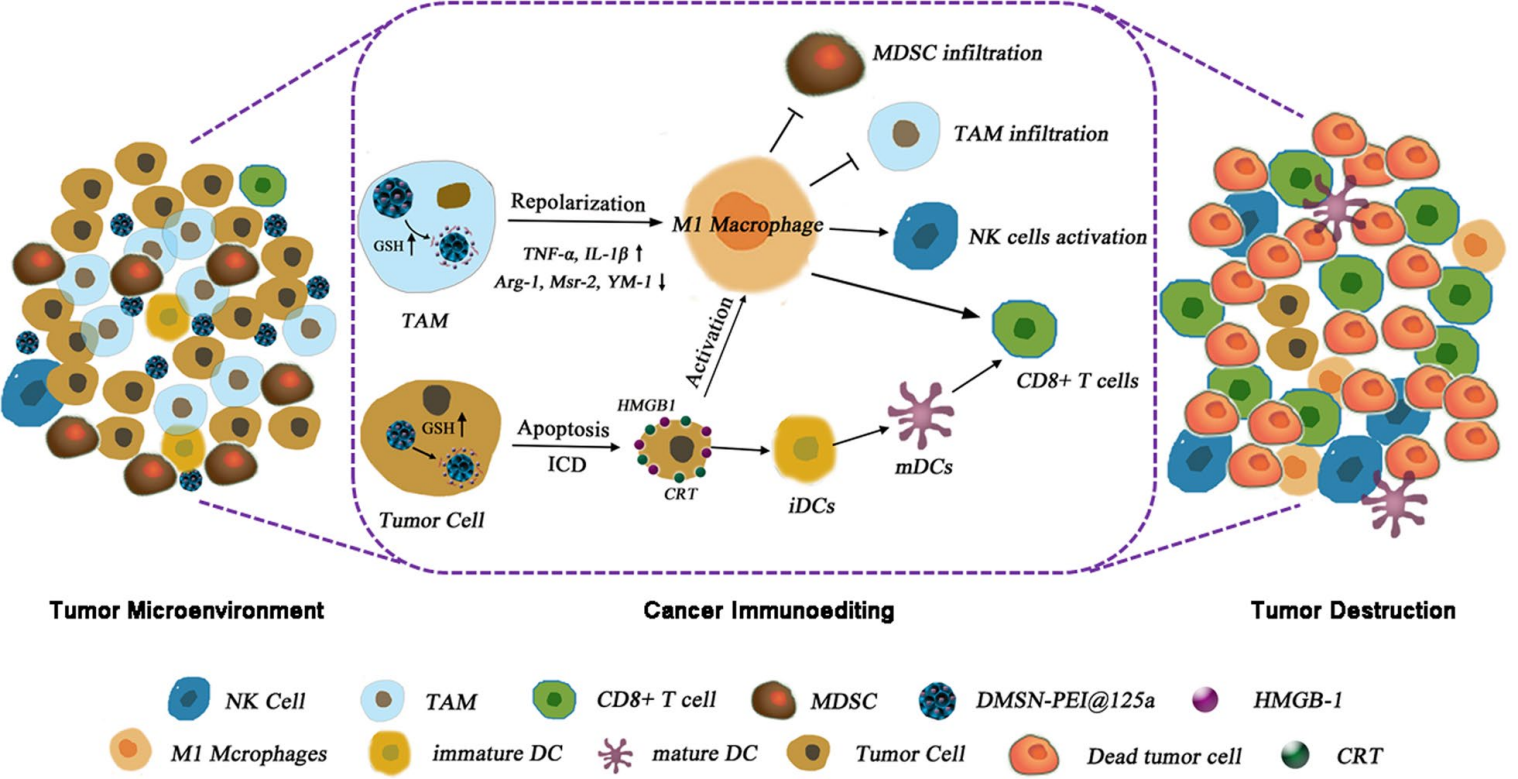
Schematic diagram of immunoediting of TC-1 microenvironment by DMSN-PEI@125a

Overall, as illustrated in Fig. 8, DMSN-PEI@125a demonstrated a superior ability to remodel the tumor microenvironment by repolarizing TAM to antitumor M1 type, facilitating apoptosis and immunogenic death of TC-1 cells, which consequently caused CD8^+^ T and NK cell proliferation, MDSCs and TAMs reduction, and tumor growth retardation through i.t. injection. However, it is worthy of note that i.t. injection limits the extensive clinical application of DMSN-PEI@125a. Compared to i.t. injection, i.v. injection is more clinically relevant, particularly for metastatic or blood tumors. We will verify the efficacy of DMSN-PEI@125a through i.v. injection in other tumor models even metastasis models in subsequent experiments.

### Biosafety of DMSN-PEI@125a in vivo

For nanoparticle-based miRNA therapy, biosafety is vital for future use in clinical treatment. Therefore, we assessed the biosafety of DMSN-PEI@125a in vivo. In the present study, mice weights of each treatment group were monitored every 3 days during the therapeutic process. As shown in Additional file 1: Fig. S5 of supporting information, all treatment groups showed similar body weight profiles compared with the control group, indicating that all these treatments when administrated via i.t. injection had no apparent side effects on mice. Furthermore, at the end of the experiment, mice were sacrificed and the serum, major organs (heart, liver, spleen, lung, and kidney) were extracted for further analysis, including blood chemistry and H&E staining. As depicted in Additional file 1: Fig. S6, HE images showed no clear signs of the histopathological abnormalities among the treatment groups compared with the control group. Meanwhile, crucial indices, including serum alanine aminotransferase (ALT), aspartate aminotransferase (AST), urea, and uric acid (UA) levels were measured and evaluated for normal liver and kidney functions. As shown in Additional file 1: Table S3 of supporting information, the total values of each index were within the reference range among all groups [41]. In general, these results suggest that DMSN-PEI@125a have a good biocompatibility in vivo.

## Conclusions

Enhancing the immune response and reversion of immunosuppression synchronously in TIME remains an immense challenge. In the present study, we constructed an intelligent DMSN-PEI@125a nanocomposite with redox-responsive gene release by fracture of disulfide bonds to accelerate tumor growth retardation by TIME remolding. DMSN-PEI loaded with miR-125a showed superior ability in cellular uptake of murine macrophages and the cervical cell line TC-1 either in vitro or in vivo. Furthermore, DMSN-PEI@125a could release the gene drug in a GSH-enriched environment. Crucially, DMSN-PEI@125a could reverse TAM to the M1 type synergistically and facilitate tumor cell apoptosis and immunogenic death, which showed tremendous potential in TIME remolding. Furthermore, we demonstrated that DMSN-PEI@125a inhibited TC-1 tumor progression, generating a tumoricidal microenvironment with CD8^+^ T and NK cell elevation, and MDSC decrease. Given the numerous merits, such as redox-responsive gene release, RNase A protection, and synergistic effect in robust remodeling of the TIME, DMSN-PEI@125a have potential for effective cancer immunotherapy.

## Supplementary Information

**Additional file 1: Figure S1.** TEM image of DMSN-PEItreated by GSH solution (10^−2^ M) for 24 h. **Figure S2.** Zeta potentials of (A) DMSN, (B) DMSN-NH_2_, (C) DMSN-s-s-COOH, (D) DMSN-PEI, (E) Histograms of Zeta potential values. **FigureS3.** (A) N_2_ adsorption/desorption isotherm (B) pore size distribution curve. **FigureS4.** miRNA release from DMSN-PEI@125a in GSH-enriched environment. **Figure S5.** Body weight of mice weight during the experimental period. **Figure S6.** HE staining of major mice organs (hearts,livers, spleens, lungs, and kidneys) at the end of the experiment. Bar = 200 μm. **Table S1. **q-PCR primers in the experiment(for mouse). **Table S2. **the specific surface area and pore volume. **Table S3.** liver or kidney parameters analysis of serum.

## Data Availability

All data generated or analysed during this study are included in this published article.

## Abbreviations

TIME: Tumor immune microenvironment
DMSN-PEI@125a: Polyethyleneimine (PEI)-modified dendritic mesoporous silica nanoparticles (DMSN) loaded with miR-125a
TAMs: Tumor associated macrophages
DAMPs: Damage-related molecular patterns
NK cells: Natural killer cells
ITM: Immunosuppressive tumor microenvironment
ICD: Immunogenic cell death
TEM: Transmission electron microscope
PDI: Polydispersity index
APC: Antigen-presenting cells
CRT: Calreticulin
HMGB-1: High mobility group box 1
H&E: Hematoxylin and eosin
ALT: Alanine aminotransferase
AST: Aspartate aminotransferase
UA: Uric acid
