# Supplementary material for: Multifunctional silica nanocomposites prime tumoricidal immunity for efficient cancer immunotherapy
Source: J Nanobiotechnology. 2021 Oct 18;19:328. doi: 10.1186/s12951-021-01073-2 (PMC8524820; doi:10.1186/s12951-021-01073-2)
Supplement: Supplementary file 1 — Additional file 1: Figure S1. TEM image of DMSN-PEItreated by GSH solution (10−2 M) for 24 h. Figure S2. Zeta potentials of (A) DMSN, (B) DMSN-NH2, (C) DMSN-s-s-COOH, (D) DMSN-PEI, (E) Histograms of Zeta potential values. FigureS3. (A) N2 adsorption/desorption isotherm (B) pore size distribution curve. FigureS4. miRNA release from DMSN-PEI@125a in GSH-enriched environment. Figure S5. Body weight of mice weight during the experimental period. Figure S6. HE staining of major mice organs (hearts,livers, spleens, lungs, and kidneys) at the end of the experiment. Bar = 200 μm. Table S1. q-PCR primers in the experiment(for mouse). Table S2. the specific surface area and pore volume. Table S3. liver or kidney parameters analysis of serum. [file 12951_2021_1073_MOESM1_ESM.docx]

**Supporting Information**

**Multifunctional silica nanocomposites prime** **tumoricidal immunity for efficient cancer immunotherapy**

Linnan Yang^1 4^ ^#^, Feng Li^1 #^, Yongsheng Cao^3^, Qiang Liu^1^, Guoxin Jing^1^, Jintong Niu^1^, Feiyue Sun^1^, Yechang Qian^2^*, Shilong Wang^1^*, Ang Li^1^*

^1^ Research Center for Translational Medicine at East Hospital, School of Life Science and Technology, Tongji University, Shanghai, P. R. China.

^2^ Department of Respiratory Disease, Baoshan District Hospital of Integrated Traditional Chinese and Western Medicine, Shanghai, P. R. China.

^3^ The Second Department of Urology, Anhui Provincial Children's Hospital, Hefei, P. R. China.

^4^ Central Laboratory, First Affiliated Hospital, Anhui Medical University, Hefei, P. R. China.

^#^These authors contribute equally to this paper

***Corresponding Author:**

Ang Li: [liang@tongji.edu.cn](mailto:liang@tongji.edu.cn)

Shilong Wang: [wsl@tongji.edu.cn](mailto:wsl@tongji.edu.cn)

Yechang Qian: [qianyechang@163.com](mailto:qianyechang@163.com)


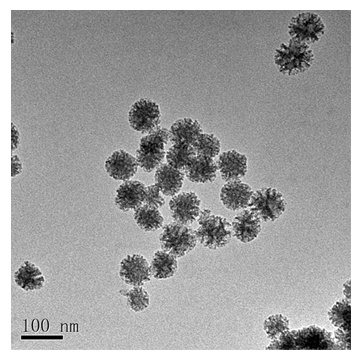


**Fig. S1** TEM image of DMSN-PEI treated by GSH solution (10^-2^ M) for 24 h.


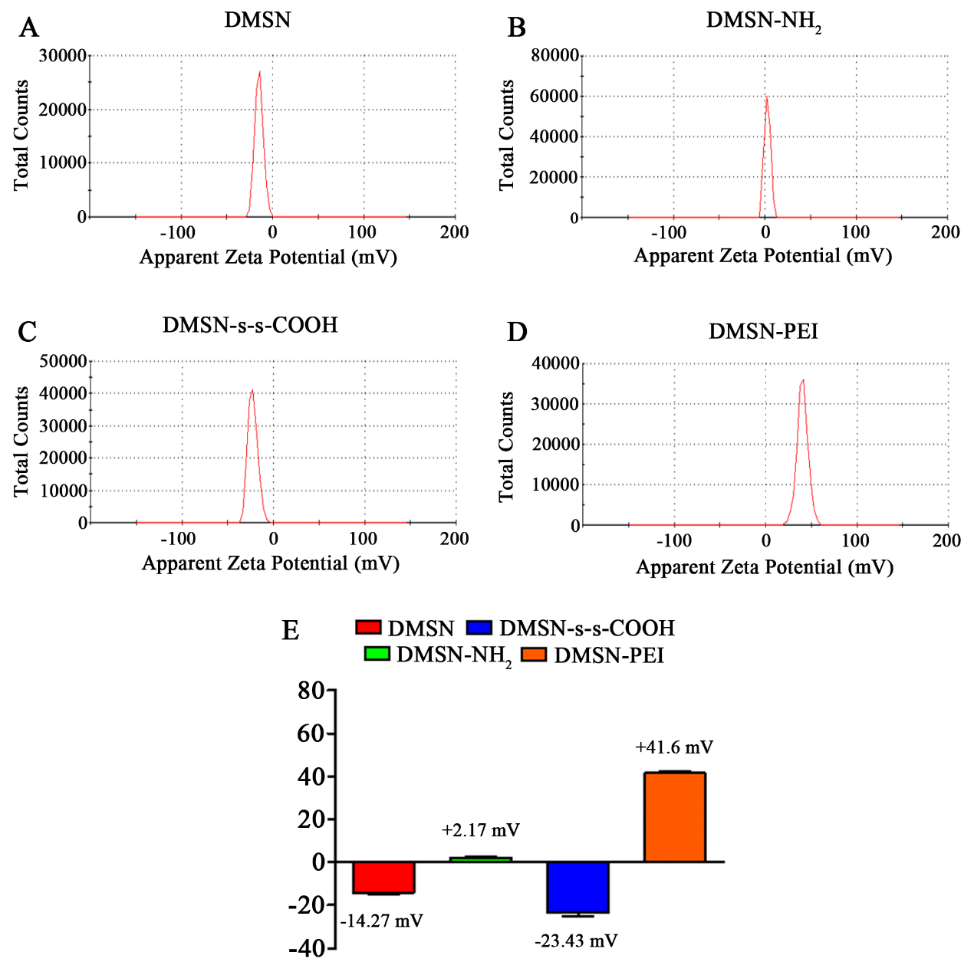


**Fig. S2** Zeta potentials of (A) DMSN, (B) DMSN-NH2, (C) DMSN-ss-COOH, (D) DMSN-PEI, (E) Histograms of Zeta potential values.


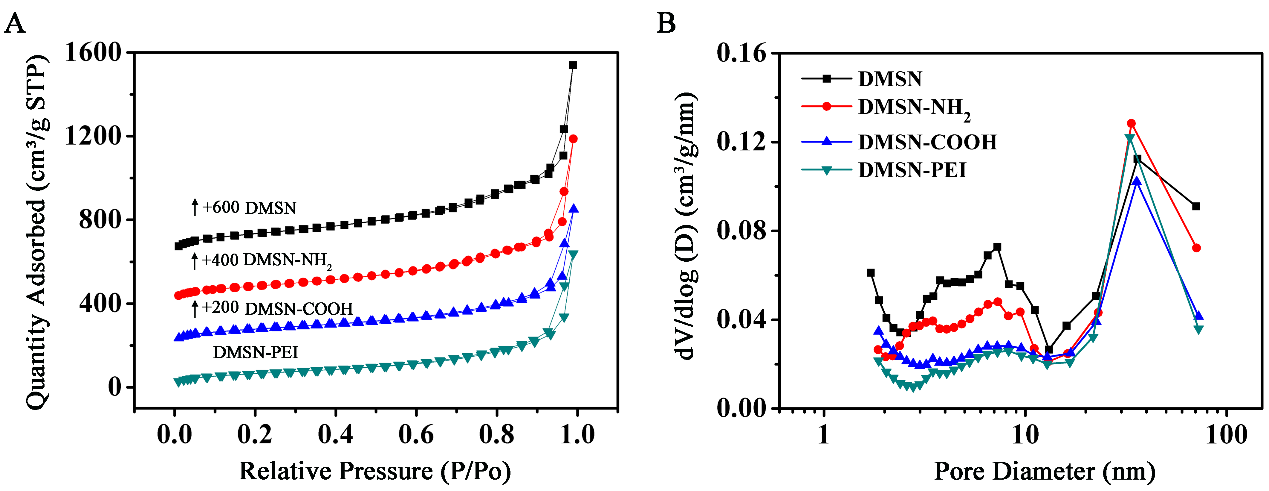


**Fig. S3** (A) N2 adsorption/desorption isotherm (B) pore size distribution curve.

***
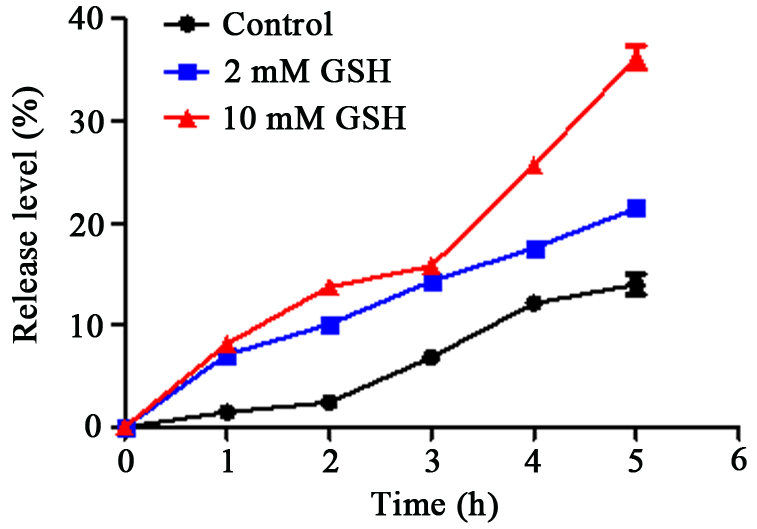
***

**Fig. S4** miRNA release from DMSN-PEI@125a in GSH-enriched environment.


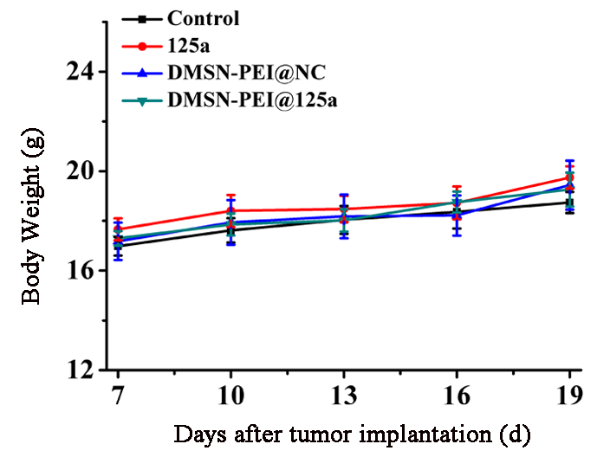


**Fig. S5** Body weight of mice weight during the experimental period.


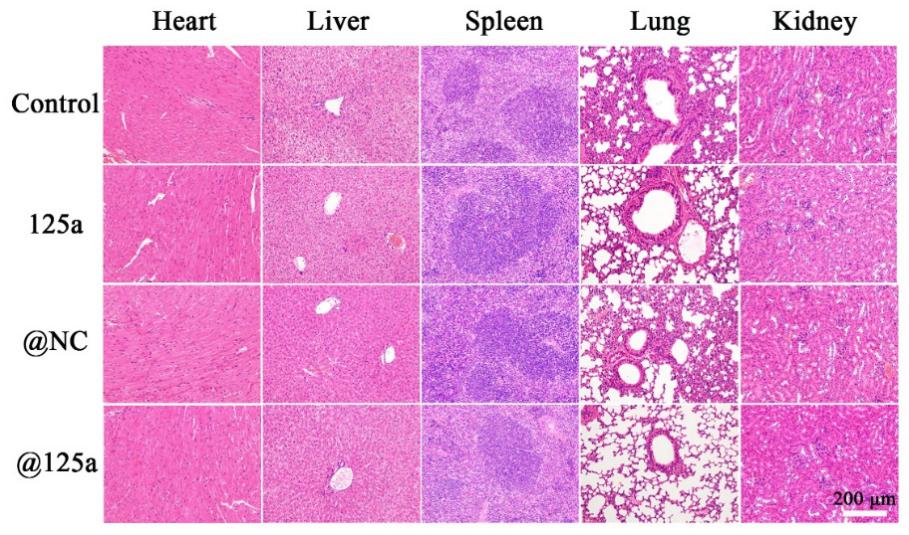


**Fig. S6** HE staining of major mice organs (hearts, livers, spleens, lungs, and kidneys). at the end of the experiment. Bar = 200 μm

**Table S1** q-PCR primer in the experiment (for mouse)

| ***Gene*** | ***Forward Primer*** | ***Reverse Primer*** |
| --- | --- | --- |
| *GAPDH* | GTGTTCCTACCCCCAATGTGT | ATTGTCATACCAGGAAATGAGCTT |
| *Arg-1* | CTCCAAGCCAAAGTCCTTAGAG | AGGAGCTGTCATTAGGGACATC |
| *TGF-β* | TGCTGCTTTCTCCCTCAACCT | CACTGCTTCCCGAATGTCTGA |
| *Msr-2* | TGCCTCTGTGCTTGCTGC | CCACTGTCACCGCGTCTT |
| *TNF-α* | CCCTCACACTCAGATCATCTTCT | GCTACGACGTGGGCTACAG |
| *IL-1β* | CCAAAAGATGAAGGGCTGCT | TCATCAGGACAGCCCAGGTC |
| *iNOS* | ATCTTTGCCACCAAGATGGCCTGG | TTCCTGTGCTGTGCTACAGTTCCG |
| *STAT3* | AGGAGTCTAACAACGGCAGCCT | GTGGTACACCTCAGTCTCGAAG |
| *Caspase-3* | GGAGTCTGACTGGAAAGCCGAA | CTTCTGGCAAGCCATCTCCTCA |
| *Caspase-9* | GCTGTGTCAAGTTTGCCTACCC | CCAGAATGCCATCCAAGGTCTC |
| *HMGB-1* | CCAAGAAGTGCTCAGAGAGGTG | GTCCTTGAACTTCTTTTTGGTCTC |
| *CRT* | AAAGGACCCTGATGCTGCCAAG | TCAGGGATGTGCTCTGGCTTGT |
| *S100-A8* | CTACTGAGTGTCCTCAGTTTGTGCAG | CCATCGCAAGGAACTCCTCGAAG |
| *S100-A9* | GCGCAGCATAACCACCATCATCG | GCCAACTGTGCTTCCACCATTTG |

**Table S2** the specific surface area and pore volume

| Sample | BET surface area (m^2^/g) | Pore volume (cm^3^/g) |
| --- | --- | --- |
| DMSN | 483.2105 | 1.4536 |
| DMSN-NH_2_ | 312.4709 | 1.2172 |
| DMSN-COOH | 290.8651 | 1.0052 |
| DMSN-PEI | 243.2834 | 0.9888 |

**Table S3** liver or kidney parameters analysis of serum

|  | Index | Ref range | Control | 125a | @NC | @125a |
| --- | --- | --- | --- | --- | --- | --- |
| **Liver parameter** | ALT (U/L) | 42-74 | 46.442 | 50.246 | 53.485 | 67.008 |
|  | AST (U/L) | 51-122 | 92.115 | 107.421 | 91.145 | 98.216 |
| **Kidney parameter** | CREA (μmol/L) | 8.8-13.2 | 9.357 | 9.907 | 10.011 | 11.069 |
|  | UA (mmol/L) | 0.1-0.7 | 0.508 | 0.631 | 0.657 | 0.665 |
